# A case report of vaping-associated sudden cardiac arrest in a young healthy patient

**DOI:** 10.1097/MS9.0000000000001907

**Published:** 2024-03-18

**Authors:** Hasaan Ahmed, Mahmoud Ismayl, Anirudh Palicherla, Joshua May, Andrew M. Goldsweig, Joseph Thirumalareddy

**Affiliations:** a Department of Medicine, Division of Internal Medicine, Creighton University School of Medicine, Omaha, Nebraska; b Department of Cardiovascular Medicine, Mayo Clinic, Rochester, Minnesota; c Department of Cardiovascular Medicine, Baystate Medical Center, Springfield, Massachusetts, USA

**Keywords:** cardiac arrest, case report, electronic cigarettes, vaping, ventricular tachycardia

## Abstract

**Introduction and importance:**

While vaping has increased significantly among young individuals, the effects of vape aerosol constituents on cardiac electrophysiological dynamics remain unknown.

**Case presentation:**

A 22-year-old female with a history of energy vaping presented with cardiac arrest. Found to have no pulse, CPR was started and an initial rhythm of ventricular tachycardia was obtained. Shock was administered with a follow-up rhythm of ventricular fibrillation. She was emergently defibrillated and entered atrial fibrillation with rapid ventricular response. Toxicology and troponins were all negative. Left heart catheterization and cardiac MRI were unremarkable. She was discharged with an external defibrillation vest and a tentative plan for outpatient electrophysiology study in the setting of negative work-up for cardiopulmonary arrest.

**Clinical discussion:**

Vaping-induced sudden cardiac arrest may be attributed to a reduction in cardiac repolarization reserve. Exposure to vegetable glycerin and propylene glycol, substances present in nearly all vape products, have been found to incite arrhythmias and disrupt cardiac conduction in animals. Acrolein, an aldehyde byproduct of glycerin, has also been found to induce arrhythmias due to autonomic dysfunction. Increased intracellular calcium concentration and free radical damage, which occur as a result of inhaling particulate matter generated from e-cigarettes, further propagates the risk of arrhythmia.

**Conclusion:**

The effects of inhaling vape aerosols remain not fully understood. While there is a perceived notion that nicotine-free aerosols may be harmless, that remains unclear. Further studies are needed to evaluate proarrhythmogenic effects and autonomic dysfunction from the various chemical substances present in vape aerosols.

## Introduction

Highlights

- Vaping continues to be popular among young nonsmoking adults.
- There remains a misconception that non-nicotine vaping is harmless.
- Vaping-induced arrhythmias may be due to reduced cardiac repolarization reserve.
- The size of inhaled particulate matter likely also promotes arrhythmias.

Vaping, which involves inhaling and exhaling aerosol content produced by an electronic cigarette, continues to increase among young adults, with an estimated 75% of vape users exclusively consuming e-cigarette products and not tobacco^1,2^. The prevalence of vape products, conventionally known as electronic cigarettes or e-cigarettes, among adolescents is propagated by a myriad of appealing flavors, targeted advertisements, and ignorance of its deleterious health consequences. Given that e-cigarettes are a relatively new entity with limited studies on long-term use, their prevalence among adolescents and young adults raises serious public health concerns. Discussion regarding the impact of non-nicotine vaping on electrophysiologic dynamics remains limited in existing literature. We present the first published case of vaping-associated sudden cardiac arrest in a young healthy adult in accordance to SCARE (Surgical CAse REport) guidelines^3^.

## Case presentation

**Figure 1:**
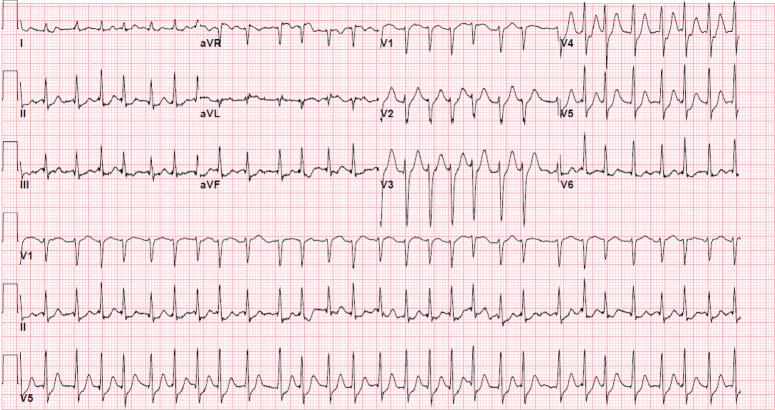
Electrocardiogram, Electrocardiogram showing atrial fibrillation with rapid ventricular response in the setting of a prolonged QTC interval, noted after emergent defibrillation.

A 22-year-old female Caucasian college-student, who engaged in active energy vaping, presented to the University Medical Center with out-of-hospital cardiac arrest via ambulance. Bystanders initially found her to be unresponsive. Resuscitation was started, and emergency services found an initial rhythm of pulseless ventricular tachycardia. She was shocked and converted to ventricular fibrillation. She was emergently defibrillated, and an ECG revealed atrial fibrillation with rapid ventricular response in the setting of a prolonged QTc interval (Fig. 1).

She was subsequently intubated. Vitals were notable for tachycardia and mild hypertension. Physical exam revealed positive withdrawal to pain, mechanical breath sounds, an irregularly irregular pulse, and no appreciable murmurs, gallops, or rubs. Her BMI was determined to be within normal limits based on her height and weight. Amiodarone, esmolol, and heparin drips were initiated.

The patient’s past medical history was unremarkable with no prior hospitalizations or major surgeries. There was no significant family history of any genetic disorders, channelopathies, or early-onset or sudden cardiac arrest. She never used any illicit or recreational drugs and consumed alcohol only during social occasions. She initially started vaping 1 year before being hospitalized and was always a non-nicotine e-cigarette user. She endorsed vaping several times daily.

Several diagnoses were considered including cardiomyopathies, valvular abnormalities, syncope, pulmonary embolism, congenital heart diseases, myocardial ischemia, and arrhythmias. Urine toxicology screen, ethanol level, and troponin were unremarkable. There were several diagnostic challenges present at the time of evaluation, such as the communication barrier between providers and the patient, as she was intubated, and the lack of truly knowing what had happened before her out-of-hospital cardiac arrest as she was found alone.

**Figure 2:**
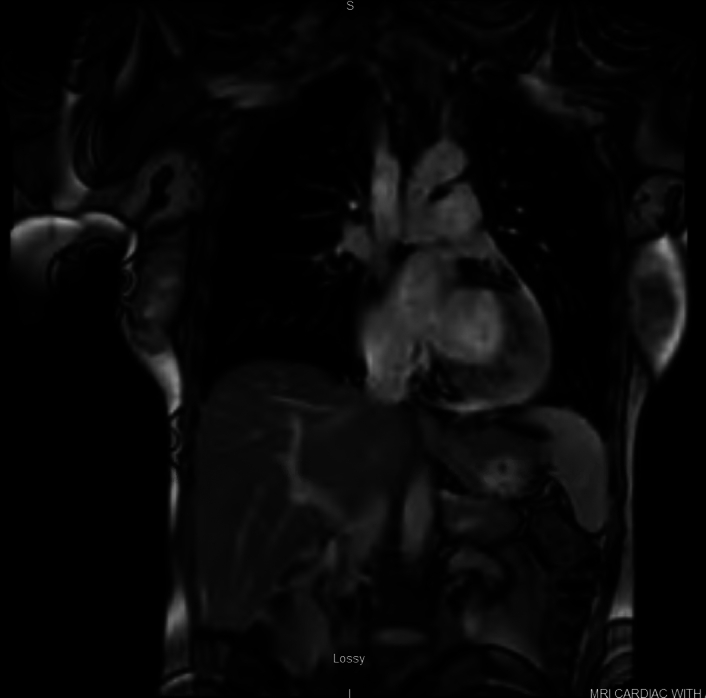
Cardiac MRI, Cardiac MRI demonstrating no evidence of myocardial scarring/fibrosis with no late gadolinium enhancement.

Chest computed tomography showed no pulmonary embolism. A transthoracic echocardiogram revealed normal biventricular function and no valvular abnormalities. Left heart catheterization, performed by the interventional cardiology team, showed angiographically normal coronary arteries. Cardiac MRI demonstrated no indication of myocardial scarring/fibrosis with no late gadolinium enhancement (Fig. 2).

The patient underwent extensive autoimmune workup which was unrevealing. Autoimmune workup consisted of an erythrocyte sedimentation rate of 1 mm/h (reference level of 0–20 mm/h), C-reactive protein of 3.22 mg/l (reference level ≤9.00 mg/l), rheumatoid factor of <10 IU/ml (reference level ≤15 IU/ml), negative antinuclear antibodies (ANA), and negative antineutrophil cytoplasmic antibodies (ANCA).

She pharmacologically cardioverted into sinus rhythm and was transitioned to metoprolol tartrate twice daily. She was extubated on day four of hospitalization. Given that she underwent extensive but unrevealing workup for sudden cardiac arrest, she was strongly counseled to refrain from vaping and was discharged with an external defibrillation vest along with a tentative plan for outpatient electrophysiology study.

**Figure 3:**
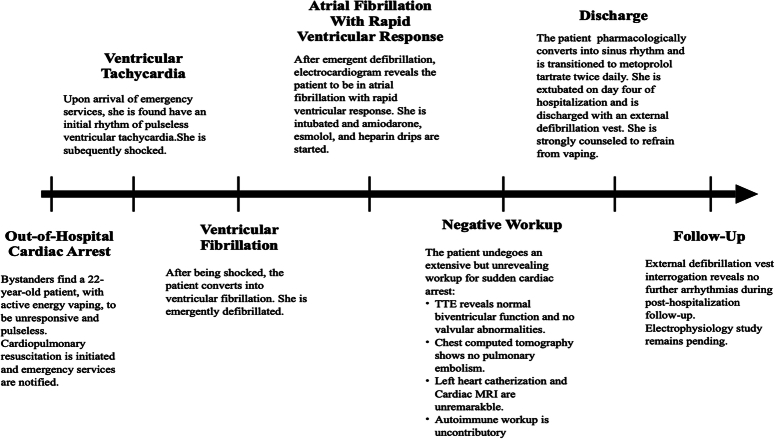
Timeline, Timeline of events highlighting the patient’s hospital course of sudden cardiac arrest.

External defibrillation vest interrogation, conducted posthospitalization, revealed no further arrhythmias. She recovered near full cognitive and physical functioning, and endorsed continued abstinence from vaping. Electrophysiology study remains pending. The patient’s perspective of her course of events (Fig. 3) was that of extreme gratitude, knowing that she survived, and significant apprehensiveness about vaping again. She will continue to be followed as a patient of the University cardiology clinic.

## Discussion

Electronic cigarettes were initially introduced as an alternative to conventional tobacco cigarettes to attract consumers to a ‘healthier’ substitute^1,2,4^. The reasoning behind the claim that electronic cigarettes are ‘healthier’ lies in the assumption that fewer toxic chemicals are being released during aerosolization than tobacco combustion. While the targeted audience of electronic cigarettes was middle-aged and elderly tobacco users, coincidentally adolescents, a majority of whom have no prior history of tobacco use, represent the largest growth market among e-cigarette users. According to the 2021 *National Health Interview Survey*, the most likely users of e-cigarettes were those between the ages of 18–24, with white non-Hispanic individuals being the most prominent racial/ethnic group among users^5^.

Electronic cigarettes contain three components: a heating element, a power source (such as a battery), and a solvent or liquid (e-liquid), which undergoes aerosolization and is ultimately inhaled^4,6^. The amount of aerosolized inhaled content is based on not only the actions of the user but also the voltage of the e-cigarette and the e-liquid ingredients. While the makeup of the inhaled aerosolized vapor varies, it generally includes a combination of a flavoring agent and a solvent, such as vegetable glycerin (VG), propylene glycol (PG), or a combination thereof, with or without nicotine^4,7^. Inhaled toxicants are also generated through the thermolysis of e-liquid content, comprised of aldehydes, volatile organic compounds, and metals^4,8^.

Given the novelty and continuous evolution of e-cigarettes, literature evaluating the electrophysiologic effects of non-nicotine vaping remains limited, with the majority being preclinical studies^4^. In a study evaluating the electrophysiologic effects of aerosols generated from nicotine-free e-cigarettes in mice, ventricular tachyarrhythmias were noted to increase significantly upon exposure to nicotine-free PG while exposure to VG aerosols elicited bradyarrhythmias and repolarization changes^9^. Given that both the sympathetic and parasympathetic nervous systems regulate heart rate, abnormalities noted in heart rate variation reflect an imbalance of autonomic functioning^4^. These outcomes suggest that there may be a risk of cardiovascular electrophysiologic disturbances resulting from autonomic abnormalities resulting in proarrhythmic states manifesting as lengthened ventricular repolarization, accelerated atrioventricular conduction, decreased heart rate variability, and increased ventricular arrhythmias^9^. Furthermore, the significant increase in ventricular tachyarrhythmias noted with PG exposure further reflects the arrhythmogenic properties of e-cigarette components, with ventricular premature beats known to be a prognostic indicator for cardiac mortality.

Exposure to acrolein, an aldehyde byproduct of glycerin, amplifies both heart rate variability and the incidence of arrhythmias in mice, with prolonged QTc intervals noted postexposure^4^. This was affirmed in a retrospective analysis of individuals who experienced sudden-onset cardiopulmonary arrest by Bains *et al*.^10^ who attributed sudden cardiac arrest, amongst vaping users, to a reduction in cardiac repolarization reserve. Of all the toxicants produced by electronic cigarettes, acrolein may be the most arrhythmogenic due to the chemosensor stimulation of TRPA1 resulting in hypersympathetic activity and autonomic dysfunction^4^. Exposure to other aldehyde subtypes has also been found to incite arrhythmias, with those present in vape flavorants noted to induce arrhythmias among mice in the absence of nicotine.

Exposure to other aldehyde subtypes has also been found to incite arrhythmias, with those present in vape flavorants noted to induce arrhythmias among mice in the absence of nicotine^4^. In a study evaluating various e-liquid flavors in human-induced pluripotent stem cell-derived cardiomyocytes by Abouassali *et al*.^11^, corrected field potential duration, an indicator of the QTc interval, and beating rate were significantly increased (*P*<0.01) after exposure to 25% of apple jax e-vapor compared to the control. There may be a heightened risk of ventricular fibrillation and ventricular tachycardia occurring in vaping users, as reflected by the increased corrected action potential duration noted. Furthermore, significant electrical alternans were appreciated in the mean action potential durations of vaped hearts (*P*<0.01) suggesting that vaping may be promoting arrhythmias through destabilizing electrophysiological dynamics.

Prior studies have noted e-cigarette users inhale a profoundly unhealthy amount of particulate matter, with particles less than 2.5 micrometers known to increase the risk of arrhythmia^4^. Tanwar *et al*. elaborates on particulate matter exposure, highlighting both direct and indirect routes of potentiating arrhythmias^4,12^. Directly, particulate matter can reach the heart through blood circulation from the lungs, resulting in increased intracellular calcium concentrations and free radical damage, amplifying the risk of arrhythmia. Indirectly, the accumulation of particulate matter in the lungs incites autonomic abnormalities and inflammatory changes, thereby resulting in vasoconstriction along with alteration in heart rate variability.

Guidelines regarding vaping continue to evolve given both its increased prevalence among young adults and emerging data regards its effects^6^. The C*enters for Disease Control and Prevention* (CDC) condemns the use of electronic cigarettes in young adults and adolescents, citing the harmful content of vaping aerosols, incidences of vaping-associated lung injury, increased risk of future substance abuse, and inhibition of brain development^13^. The CDC strongly recommends a multimodal approach of education, awareness, recognition, and counseling to reduce vaping among young individuals^14^. Furthermore, the CDC urges caution with the assumption that e-cigarettes are healthier than traditional smoking, as there remain significant knowledge gaps regarding its long-term effects^6,13^. The *American Heart Association* strongly urges further investigation into the use of vaping given that e-cigarettes are not regulated as medications, bypassing the need for human safety studies, with significant uncertainty regarding short-term and long-term health impacts^6^. Furthermore, prior studies on electronic cigarette users have primarily evaluated individuals who were conventional smokers or former smokers, limiting their applicability to young, nonsmoking, vaping users.

Current guidelines recommend e-cigarette users quit vaping, citing the lack of demonstrated evidence in helping tobacco users quit smoking with the concern that vaping may be normalizing smoking among young users^15^. Additionally, preventative benefits of quitting vaping include enhancements to sensory functions and improved physique, along with psychosocial benefits such as increased confidence, decreased stress, and reduced anxiety^16^. Despite the widespread use of e-cigarettes, the desire to quit vaping among adolescents and young adults continues to increase, as reflected in a retrospective study by Cha *et al*., which noted health concerns being the most prevalent reason cited by young individuals who quit vaping, propagated by stricter regulations of e-cigarettes and heightened attention of vaping-associated adverse health effects^17^.

## Conclusion

Electronic cigarettes continue to be popular among young individuals, perpetuated by the misconception that non-nicotine vaping is harmless. Our case of vaping-associated sudden cardiac arrest in an otherwise healthy 22-year-old adds to the current literature by expanding upon the impact of vape aerosol constituents on propagating arrhythmias and the need for further studies to investigate the electrophysiologic effects of vaping.

## Ethical approval

Ethics clearance was not necessary as this was a case report. There was no research study conducted and Institutional Review Board approval was not needed.

## Consent

Written informed consent was obtained from the patient for publication of this case report and accompanying images. A copy of the written consent is available for review by the Editor-in-Chief of this journal on request.

## Sources of funding

We, the authors, have nothing to declare in this category as it is not applicable.

## Author contribution

H.A., M.I., A.P., J.M., A.G., and J.T.: conceptualization; H.A., M.I., A.P., J.M., and A.G.: writing – original draft; H.A., M.I., A.P., J.M., A.G., and J.T.: writing – review and editing; H.A., M.I., A.P., and J.M.: investigation; A.G. and J.T.: supervision.

## Conflicts of interests disclosure

The authors declare that they have no known competing financial interests or personal relationships that could have appeared to influence the work reported in this paper.

## Research registration unique identifying number (UIN)

1. Name of the registry: Research registry.
2. Unique identifying number or registration ID: researchregistry10005.
3. Hyperlink to your specific registration: https://researchregistry.knack.com/researchregistry#user-researchregistry/registerresearchdetails/65c975794455e70027053eba/.

## Guarantor

Hasaan Ahmed M.D.

## Data availability statement

We, the authors, have nothing to declare in this category as it is not applicable.

## Provenance and peer review

Not commissioned, externally peer-reviewed.

## References

[1] American Heart Association. Current evidence identifies health risks of e-cigarette use; long-term research needed. American Heart Association. Published 17 July 2023. https://newsroom.heart.org/news/current-evidence-identifies-health-risks-of-e-cigarette-use-long-term-research-needed

[2] McCauley DM Gaiha SM Lempert LK. Adolescents, young adults, and adults continue to use e-cigarette devices and flavors two years after fda discretionary enforcement. Int J Environ Res Public Health 2022;19:8747. 35886599 10.3390/ijerph19148747 PMC9322506

[3] Sohrabi C Mathew G Maria N. The Scare 2023 guideline: updating consensus surgical case report (SCARE) guidelines. Int J Surg 2023;109:1136–1140. 37013953 10.1097/JS9.0000000000000373 PMC10389401

[4] Jones CV Wallace MJ Bandaru P. E-cigarettes and arrhythmogenesis: a comprehensive review of pre-clinical studies and their clinical implications. Cardiovasc Res 2023;119:2157–2164. 37517059 10.1093/cvr/cvad113 PMC10578912

[5] Kramarow E Elgaddal N. Current electronic cigarette use among adults aged 18 and over: United States, 2021. NCHS Data Brief 2023:1–8. https://www.cdc.gov/nchs/data/databriefs/db475.pdf 37486729

[6] Rose JJ Krishnan-Sarin S Exil VJ. Cardiopulmonary impact of electronic cigarettes and vaping products: a scientific statement from the American Heart Association. Circulation 2023;148:703–728. 37458106 10.1161/CIR.0000000000001160

[7] Tokle R Brunborg GS Vedøy TF. Adolescents’ use of nicotine-free and nicotine e-cigarettes: A longitudinal study of vaping transitions and vaper characteristics. Nicotine Tob Res 2021;24:400–407. 10.1093/ntr/ntab192 PMC8842395 34546348

[8] Breland A Soule E Lopez A. Electronic cigarettes: what are they and what do they do. Ann N Y Acad Sci 2016;1394:5–30. 26774031 10.1111/nyas.12977 PMC4947026

[9] Carll AP Arab C Salatini R. E-cigarettes and their lone constituents induce cardiac arrhythmia and conduction defects in mice. Nat Commun 2022;13:6088. 36284091 10.1038/s41467-022-33203-1 PMC9596490

[10] Bains S Garmany R Gao X. PO-704-02 Vaping-associated sudden death in the young. Heart Rhythm 2022;19:S450.

[11] Abouassali O Chang M Chidipi B. In vitro and in vivo cardiac toxicity of flavored electronic nicotine delivery systems. Am J Physiol-Heart Circulatory Physiol 2021;320:H133–H143. 10.1152/ajpheart.00283.2020 PMC7847071 33216635

[12] Tanwar V Adelstein JM Wold LE. Double trouble: combined cardiovascular effects of particulate matter exposure and COVID-19. Cardiovasc Res 2020;117:85–95. 10.1093/cvr/cvaa293 PMC7665323 33084879

[13] CDC. Electronic Cigarettes. Centers for Disease Control and Prevention. Published 1 March 2019. https://www.cdc.gov/tobacco/basic_information/e-cigarettes/index.htm

[14] CDC. Protecting Young People From E-cigarettes. Published 20 July 2021. www.cdc.govhttps://www.cdc.gov/tobacco/features/back-to-school/index.html

[15] What You Need to Know About Vaping. Accessed 14 January 2024. www.heart.orghttps://www.heart.org/en/healthy-living/healthy-lifestyle/quit-smoking-tobacco/what-you-need-to-know-about-vaping#:~:text=The%20American%20Heart%20Association%20recommends

[16] *Benefits of Quitting Vaping or Smoking.* https://www.quithq.initiatives.qld.gov.au/__data/assets/pdf_file/0020/119711/Fact-Sheet-Benefits-of-Quitting-Vaping-or-Smoking.pdf

[17] Cha S Amato MS Papandonatos GD. Changes over time in reasons for quitting vaping among treatment-seeking young people from 2019 to 2022. Addictive Behaviors Rep 2024;19:100521. 10.1016/j.abrep.2023.100521 PMC10713839 38094667

